# Prescribed Renoprotective Chinese Herbal Medicines Were Associated with a Lower Risk of All-Cause and Disease-Specific Mortality among Patients with Chronic Kidney Disease: A Population-Based Follow-Up Study in Taiwan

**DOI:** 10.1155/2017/5632195

**Published:** 2017-07-17

**Authors:** Chuan-Fa Hsieh, Huan-Cheng Chang, Song-Lih Huang, Chien-Lung Chen, Wei-Ta Chen, Chen-Chang Yang

**Affiliations:** ^1^Department of Medical Education and Research, Taiwan Landseed Hospital, No. 77, Guangtai Rd., Pingzhen Dist., Taoyuan 32449, Taiwan; ^2^Center for General Education, Hsin Sheng College of Medical Care and Management, No. 418, Zhongxing Rd., Longtan Dist., Taoyuan 32555, Taiwan; ^3^Division of Nephrology, Department of Medicine, Taiwan Landseed Hospital, No. 77, Guangtai Rd., Pingzhen Dist., Taoyuan 32449, Taiwan; ^4^Department of Health Care Management, Chang Gung University, No. 259, Wenhua 1st Rd., Guishan Dist., Taoyuan 33302, Taiwan; ^5^Institute of Public Health & Department of Public Health, School of Medicine, National Yang-Ming University, No. 155, Sec. 2, Linong St., Taipei 11221, Taiwan; ^6^Institute of Systems Biology and Bioinformatics, Department of Biomedical Sciences and Engineering, National Central University, No. 300, Zhongda Rd., Zhongli District, Taoyuan 32001, Taiwan; ^7^Department of Chinese Medicine, Taiwan Landseed Hospital, No. 77, Guangtai Rd., Pingzhen Dist., Taoyuan 32449, Taiwan; ^8^Institute of Environmental & Occupational Health Sciences, School of Medicine, National Yang-Ming University, No. 155, Sec. 2, Linong St., Taipei 11221, Taiwan; ^9^Division of Clinical Toxicology & Occupational Medicine, Department of Medicine, Taipei Veterans General Hospital, No. 201, Sec. 2, Shipai Rd., Taipei 11217, Taiwan

## Abstract

Chinese herbal medicines (CHMs) containing aristolochic acid (AA) are associated with chronic kidney disease (CKD), but some prescribed CHMs have been shown to possess renoprotective effects. We conducted a nationwide retrospective cohort study to delineate the role of prescribed CHMs on the CKD progression. Renoprotective CHM (RPCHM) was defined if a CHM contained dong chong xia cao (*Cordyceps sinensis *(Berk.) Sacc.), da huang (*Rheum palmatum *L), huang qi* (Astragalus membranaceus)*, dan shen (*Salvia miltiorrhiza *Bge.), and dong quai (*Angelica sinensis *(Oliv.) Diels) or belonged to specific mixture herbal formulations (Yishen capsule, Saireito, or Wen Pi Tang). Subjects who had ever used AA-containing CHMs, had cancer or HIV prior to CKD diagnosis, or died within the first month of CKD diagnosis were excluded. A total of 11,625 patients were eligible subjects. The adjusted hazard ratio (aHR) for all-cause mortality was 0.6 (*p* < 0.001) and 0.6 (*p* = 0.013) among subjects receiving RPCHMs containing* Angelica sinensis *and those receiving other RPCHMs, respectively. For CKD-related mortality, the aHR among subjects receiving RPCHMs containing* Angelica sinensis *was 0.6 (*p* = 0.025). The use of specific RPCHMs, especially those that contained* Angelica sinensis*, was associated with a lower risk of mortality among CKD patients.

## 1. Introduction

Chinese herbal medicine (CHM) is one of the alternative therapies that are commonly used worldwide. It is estimated that approximately 20% of adults in the United States are taking herbal medicine [1], and the global use of herbal medicine products has increased significantly [2]. However, many studies have found that certain CHMs, primarily those that contain aristolochic acid (AA), are associated with acute renal failure, urinary tract cancer [3, 4], and increased risk of chronic kidney disease (CKD) [5]. This has led to the misnomer of “Chinese herb nephropathy” in some English literature [6]. However, not all CHMs are nephrotoxic.

In fact, some CHMs such as dong quai (*Angelica sinensis *(Oliv.) Diels), huang qi* (Astragalus membranaceus)*, and dong chong xia cao (*Cordyceps sinensis* (Berk.) Sacc.) have been shown to possess beneficial effects in the treatment of patients with CKD or to slow the progression of CKD in animal experiments [7, 8].

Taiwan is one of the countries with the highest incidence and prevalence of end-stage renal disease (ESRD) in the world [9]. The use of CHMs is fairly common in this country, even among those patients with CKD and subsequent ESRD [10, 11]. While most prior studies in Taiwan revealed a positive association between the use of CHMs and the risk of CKD [3, 5, 12], recent studies have demonstrated the potential protective effects of CHMs on the progression of CKD [11, 13, 14]. The aim of this study therefore is to delineate the possible beneficial role of various non-AA prescribed CHMs on the progression of CKD among patients with incident CKD by employing a retrospective cohort study of CKD patients in Taiwan.

## 2. Materials and Methods

### 2.1. Study Population

The mandatory National Health Insurance (NHI) program in Taiwan was implemented in 1995 and covered more than 96% of the 22.5 million Taiwanese as of 2002. All treatment information of NHI enrollees, including medical diagnoses according to the International Classification of Diseases, Ninth Revision (ICD-9-CM), hospital admissions, emergency department and outpatient visits, and the prescription of both conventional medicines and CHMs, is systematically collected and entered into the National Health Insurance Research Database (NHIRD) that is accessible for research purposes.

We employed the Longitudinal Health Insurance Database (LHID2000), which was created by the NHI Bureau in 2000 and comprises one million randomly sampled enrollees who were insured under the NHI program in 2000. LHID2000 contains follow-up medical information for all NHI enrollees included in the database spanning from 1997 to 2008, and many papers have been published using this database [14, 15]. All records were anonymized and deidentified prior to statistical analysis.

### 2.2. Eligible Participants and Duration of Follow-Up

Using the LHID2000, we searched for the following ICD-9-CM diagnostic codes for all outpatient visits or hospital admissions to identify potential cases of CKD: 581-582, 585–589, 250.4, 274.1, 403, and 404. Because A codes were also used in Taiwan before 2002, we included the following codes in the identification of CKD: A350, A359.09, A359.10, and A358.27. CKD patients were defined as having had more than three consecutive outpatient visits (which is the same as approximately 3 months of follow-up) or hospitalized due to CKD. We selected these codes after considering the definition of CKD in several previously published papers that also employed the NHIRD [11, 12, 16, 17].

To ensure that only first-time diagnosis of CKD was included, we excluded subjects with a diagnosis of CKD between 1997 and 1999. We also excluded patients with preexisting cancer or human immunodeficiency virus (HIV) infection. Subjects without Taiwanese citizenship were also excluded, as well as those who had ever received CHMs containing AA because of its well-known nephrotoxicity and carcinogenicity [4, 12]. Finally, to minimize the possibility of confounding by contraindication, we excluded patients who died within one month after the diagnosis of CKD because they were less likely to receive CHMs due to their severe underlying diseases or even moribund state [11].

The following AA-containing CHMs were identified using the standards for prescription recommended by the Committee on Chinese Medicine and Pharmacy in Taiwan: Ma-Dou-Ling* (Fructus aristolochiae)*, Tian-Xian-Teng* (Caulis aristolochiae)*, Xi-Xin* (Asarum heterotropoides)*, Guan-Mu-Tong* (Aristolochia manshuriensis)*, Guang-Fangchi* (A. fangchi)*, Qing-Mu-Xiang* (Radix aristolochiae)*, Mu-Tong* (Akebia *sp.), Fangchi* (Stephania *sp.), and Mu-Xiang* (Radix aucklandiae)* [12, 17].

The primary outcomes of this study included death attributable to all-cause or CKD-related diseases (defined by the aforementioned ICD-9-CM codes of CKD). To allow for at least one year of follow-up, all diagnoses of CKD had to be made before December 31, 2007. Subjects were followed from the date of first CKD diagnosis until death, loss of follow-up, or December 31, 2008, whichever came first.

### 2.3. Assessment of Exposure to Potentially Renoprotective Chinese Herbal Medicines (RPCHMs)

In Taiwan, all CHMs prescribed by licensed Chinese medicine physicians are eligible for reimbursement by the NHI Bureau. To assess the potential effects of RPCHMs, we identified eight CHMs reimbursed by the NHI Bureau that have been shown to have beneficial effects on renal function in clinical and animal studies, namely* Cordyceps sinensis*, da huang (*Rheum palmatum *L.),* Astragalus membranaceus*, dan shen (*Salvia miltiorrhiza *Bge.),* Angelica sinensis*, Saireito (Chai-Ling-Tang, a mixture herbal formulation) [18, 19], Yishen capsule (an encapsulated herbal formulation) [20], and WenPi Tang (a mixture herbal formulation) [21]. Because these CHMs might be prescribed as single constituents or compound CHM formulations, it was extremely difficult to calculate the exact dose of each CHM. Therefore, we categorized the treatment duration into nonuse and use of RPCHM. All RPCHMs use was further treated as a time-dependent variable to avoid immortal time bias [22].

### 2.4. Assessment of Potential Confounders

We collected the following variables to control the effects of potential confounders, namely, age; gender; comorbidities prior to CKD diagnoses, including hypertension, diabetes mellitus (DM), and cardiovascular disease (CVD); prior hospitalization (for any reason); use of nonsteroidal anti-inflammatory drugs (NSAIDs) and analgesics and the use of erythropoietin therapy. We classified age into four groups [3]: less than 40, 40–55, 56–70, and greater than 70 years.

The diagnosis of comorbidities was determined by searching for three consecutive outpatient visits or any hospitalization for the ICD-9-CM codes within three years before the CKD was diagnosed. Charlson's Comorbidity Index (CCI) was also employed to evaluate the overall disease burden of various comorbidities on each study subject [23]. The definitions of comorbidities other than hypertension, DM, and CVD were similar to those of the aforementioned diseases. The records of any hospitalization within three years prior to the diagnosis of CKD were treated as a potential confounder in data analysis as well.

Habitual smoking and drinking are potential confounders, but no data is available in LHID2000. To control for their confounding effects, we employed the diagnosis of chronic obstructive pulmonary disease (COPD) and alcoholic liver diseases as surrogates for habitual smoking and drinking, respectively. Moreover, we evaluated the study subjects' socioeconomic status based on their monthly wage. According to the quartile distribution of subjects' monthly wages, study subjects were classified into three groups: monthly wage lower than 15,840 New Taiwan dollars (NTD, equivalent to approximately 500 United States dollars [USD], the minimum monthly wage of full-time employees in Taiwan during the study period), between NTD 15,841 and 19,200 (approximately USD 600), and more than NTD 19,200.

We also assessed the dose of NSAIDs, analgesics, erythropoietin, and vitamin D supplements because these drugs are likely to confound the relationship between the use of RPCHMs and the risk of mortality in patients with CKD. The cumulative dose of each drug given to the subjects was then calculated by the number of prescribed pills. For NSAIDs and analgesics, we divided subjects by the annual total number of pills received within the previous year into ≦104 and >104 pills [24].

Erythropoietin was used as a surrogate marker of the severity of CKD in Taiwan because its use is strictly limited by the NHI Bureau to ESRD patients who have a hematocrit (Hct) value lower than 28% or CKD patients with serum creatinine over 6 mg/dL and Hct value less than 28%. The use of erythropoietin and vitamin D supplements was classified as use or nonuse in this study.

### 2.5. Statistical Analysis

Log-rank test and Holm-Sidak multiple comparisons were used to examine the survival rates between different RPCHM treatment groups. Furthermore, a Cox proportional hazards regression model was used to evaluate the association between potential risk factors and CKD subjects' survival rate. All variables with a *p* value less than 0.05 in univariate analyses were entered into the multivariate analysis, and a stepwise procedure was used to eliminate those variables that became insignificant. In addition, to determine whether the association between RPCHM use and survival status varied across different subgroups, we conducted subgroup analyses by stratifying subjects by age, gender, DM, hypertension, use of NSAIDs and analgesics, use of erythropoietin therapy, and prior hospitalization. All statistical analyses were performed with the SAS statistical package version 9.1.3 (SAS Institute, Inc., Cary, NC, USA). A *p* value less than 0.05 was considered statistically significant. The study protocol of this retrospective cohort study was approved by the Institutional Review Board (IRB) of Landseed Hospital (Landseed IRB-12-23).

## 3. Results

**Figure 1:**
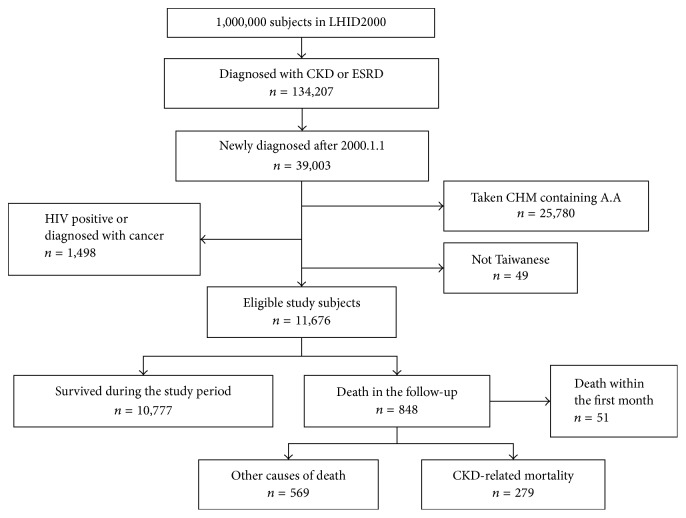
Patients' selections flow chart. AA: aristolochic acid; CHM: Chinese herbal medicines; CKD: chronic kidney disease; ESRD: end-stage renal disease; HIV: human immunodeficiency virus.

Within the database, 39,003 patients were found to have incident CKD after January 1, 2000. Among them, 25,780 patients had ever received AA-containing CHMs and were thus excluded. Forty-nine patients were of a foreign nationality, and 1,498 patients had also been diagnosed with cancer or HIV. Moreover, 51 patients died within 1 month of the CKD diagnosis. After excluding these patients, a total of 11,625 CKD patients were eligible for further analysis (Figure 1).

The mean age of the study subjects was 55.1 ± 20.9 years. A total of 848 patients died during the follow-up period, including 279 (33%) CKD-related deaths. Older age was associated with an increased risk of mortality (Table 1).

Deaths occurred more frequently in patients with various comorbidities, including hypertension, DM, CVD, higher CCI score, alcoholic liver disease, and COPD. The use of more than 104 pills of NSAIDs or analgesics per year after the diagnosis of CKD was also associated with all-cause mortality, but not with CKD-related mortality. Receipt of erythropoietin therapy was correlated with an increased risk of both all-cause and CKD-related mortality (Table 1).

**Figure 2:**
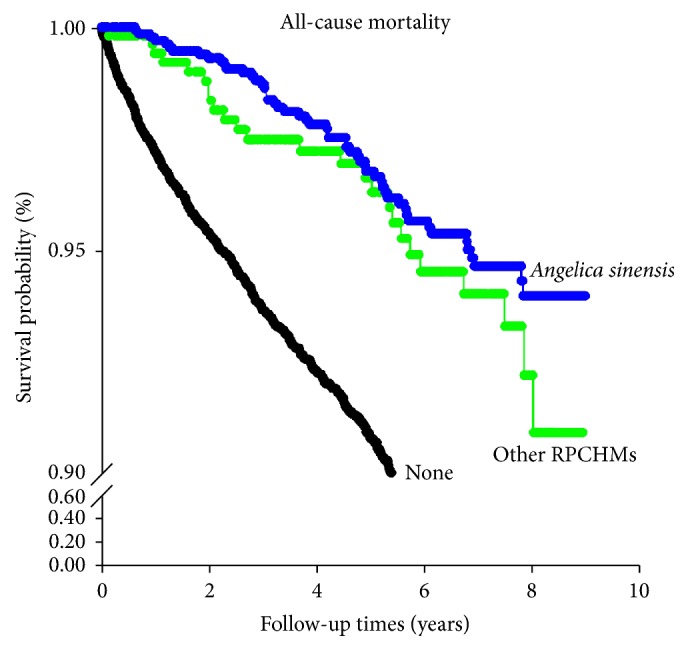
Survival curve for all-cause mortality among 11,625 patients with chronic kidney disease. RPCHM: renoprotective Chinese herbal medicines. Log-rank test (Holm-Sidak method):* Angelica sinensis* versus none, *p* < 0.001;* Angelica sinensis* versus other RPCHMs, *p* = 0.263; other RPCHMs versus none, *p* < 0.001.

In the analysis of RPCHMs, we initially sought to analyze the association between the use of each RPCHM and the risk of mortality after the diagnosis of CKD. However, only 1,919 subjects had RPCHMs prescribed after being diagnosed with CKD, and most of them (*n* = 1,381; 72%) received RPCHMs that contain* Angelica sinensis*. Therefore, we combined those patients who did not receive* Angelica sinensis* after the diagnosis of CKD into a group to allow more meaningful statistical analyses. The use of RPCHMs after the diagnosis of CKD was eventually classified into nonuse of RPCHMs, use of RPCHMs containing* Angelica sinensis*, and use of other RPCHMs. Use of RPCHMs before the diagnosis of CKD was associated with a low risk of all-cause mortality in bivariate analysis, whereas RPCHM use after the diagnosis of CKD was inversely correlated with both all-cause and CKD-related mortality (Table 1). Further analysis of the survival curve revealed that the use of RPCHMs (no matter containing* Angelica sinensis* or not) was generally associated with a lower risk of all-cause mortality compared to nonusers (Figure 2).

**Figure 3:**
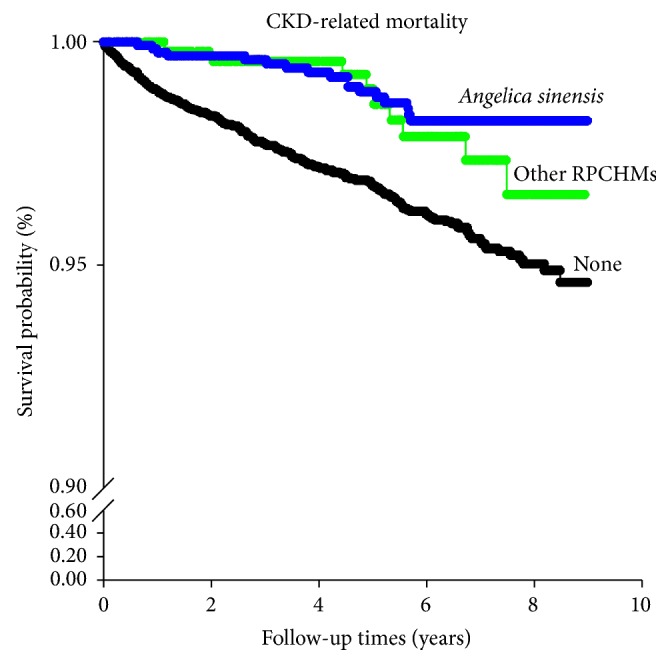
Survival curve for chronic kidney disease related mortality among 11,625 patients with chronic kidney disease. RPCHM: renoprotective Chinese herbal medicines. Log-rank test (Holm-Sidak method):* Angelica sinensis* versus none, *p* < 0.001;* Angelica sinensis* versus other RPCHMs, *p* = 0.064; other RPCHMs versus none, *p* = 0.351.

In contrast, only subjects who had received* Angelica sinensis* exhibited a better survival in CKD-related mortality than nonusers (Figure 3).

After controlling for potential confounding variables, the adjusted hazard ratio (aHR) for the use of other RPCHMs was 0.6 (95% confidence interval [CI] 0.4–0.9, *p* = 0.013), whereas the aHR for the use of* Angelica sinensis* was 0.6 (95% CI 0.4–0.8, *p* < 0.001) (Table 2). The relation between the use of* Angelica sinensis* and a lower risk of CKD-related mortality in bivariate analysis did not change after controlling for confounding variables with the aHR being 0.6 (95% CI 0.4–0.9, *p* = 0.025) (Table 2).

We also conducted subgroup analyses to see whether the findings varied across subgroups. Although the HR estimates were not available for certain subgroups because of limited sample size, the inverse association between the use of RPCHMs containing* Angelica sinensis* and the risk of mortality among CKD patients persisted across various subgroups. Nevertheless, the use of* Angelica sinensis *was not associated with reduced mortality in patients required erythropoietin therapy and use of NSAID or analgesics over 104 pills per year (Table 3).

## 4. Discussion

CKD is a worldwide public health problem and is recognized as a risk factor for cardiovascular events, hospitalization, and all-cause mortality [5, 25, 26]. The therapeutic strategies for treating CKD are generally aimed at ameliorating the progression of CKD and preventing complications. Previous studies found that the use of AA-containing or nonprescribed CHMs might lead to CKD or ESRD [10, 12]. Therefore, although animal and clinical studies have demonstrated potential renoprotective effects of certain CHMs [18], most physicians tend to persuade patients with CKD to stop taking CHMs to prevent further deterioration of their renal function. A recent study however showed that patients with CKD who used prescribed CHMs exhibited a reduced risk of ESRD [14]. In a pilot study, we also found that patients with CKD in Taiwan who started to receive non-AA prescribed CHMs after the diagnosis of incident CKD had a lower risk of mortality compared with nonusers of non-AA prescribed CHMs (aHR 0.6; 95% CI 0.4 to 0.7) [11]. Moreover, patients with CKD who had received non-AA prescribed CHMs both prior to and after the diagnosis of CKD also had a lower risk of mortality than nonusers (aHR 0.6; 95% CI 0.5 to 0.8) after the onset of CKD. In another study, the use of CHMs was independently associated with a decreased risk of renal failure among type 2 diabetic patients (aOR = 0.7, 95% CI 0.6–0.8) (aOR = 0.7, 95% CI 0.6–0.8) [11].

In this population-based follow-up study, we found that most prescribed RPCHMs were inversely associated with the risk of all-cause mortality among patients with CKD. Moreover, the use of RPCHMs containing* Angelica sinensis* after the diagnosis of CKD was associated with a lower risk of CKD-related mortality. The possible renoprotective effects of RPCHMs largely persisted in subgroup analyses, but the seemingly beneficial effect disappeared among patients who used NSAIDs or analgesics more than 104 pills per year or who already had severe CKD, as indicated by the receipt of erythropoietin therapy.

*Angelica sinensis* is commonly used in CHM to enrich blood and promote blood circulation [27]. It has been found to have anti-inflammatory effects and can improve immune function by increasing the expression of cytokines in splenocytes and the production of interleukin-6 and interferon-*γ* of activated macrophages, helper T cells, and natural killer cells [28]. A previous study reported that the combined use of* Angelica sinensis *and other CHMs such as* Astragalus membranaceus* and* Astragali radix* exerted antifibrosis effects and were associated with improvements in ischemic microvasculature and renal interstitial fibrosis [29].

The major ingredients of* Angelica sinensis* include ligustilide, n-butylidene phthalide, ferulic acid, nicotinic acid, and sesquiterpenoid compounds [30]. These ingredients have been found to possess analgesic, anti-inflammatory, immunomodulatory, and even anticancer effects [31, 32]. Among them, ligustilide is the major active component of* Angelica sinensis* and has many pharmaceutical effects such as vasodilatation [33] and inhibition of the proliferation of vascular smooth muscle cells to retard vascular sclerosis [34]. Therefore,* Angelica sinensis* may have beneficial effects on the progression of CKD and subsequently reduce the mortality of CKD patients.

In addition to* Angelica sinensis*, the use of other RPCHMs was associated with a lower risk of all-cause mortality. Among the other RPCHMs evaluated in this study,* Astragalus membranaceus* is also commonly used in CHM. It is usually prescribed in conjunction with* Angelica sinensis*, a combination therapy that has been found to inhibit renal tubulointerstitial fibrosis in rats with obstructive nephropathy [19, 35].

Similarly, Shahed et al. found that* Cordyceps sinensis* exhibited potent antioxidant activity and protected the kidneys by inhibiting mesangial cell proliferation [36]. In a clinical trial, long-term use of* Cordyceps sinensis *for up to 3 years led to reduced levels of creatinine and blood urea nitrogen (BUN) in patients with lupus nephritis [37]. In another trial,* Cordyceps sinensis *displayed a protective role in aminoglycoside induced nephrotoxicity in the elderly [38].

The use of 6 to 9 grams of* Rheum palmatum* per day in patients with CKD was also found to effectively delay the progression of CKD to ESRD [39].* Salvia miltiorrhiza* contains at least seven phenolic compounds with strong antioxidant activities that were effective in reducing BUN and eventual renoprotection in rats with impaired renal function [40].* Salvia miltiorrhiza* was also shown to improve platelet function and regulation of coagulation in dialysis patients [41].

Yishen capsule was effective in reducing the production of proteinuria in patients with chronic glomerulonephritis [42]; improving serum creatinine, BUN and cholesterol; and consequently ameliorating the progression to ESRD [20]. In addition, many Japanese studies have indicated that Saireito and Wen Pi Tang could lower the levels of serum creatinine, urine protein, and systolic blood pressure, thus making them effective in delaying the progression of CKD to ESRD [21, 43, 44].

We did observe an inverse association between the use of RPCHMs other than* Angelica sinensis* and the risk of all-cause mortality in this study. However, we were unable to ascertain which of the other RPCHMs contributed to the beneficial effects on the mortality of patients with CKD because of the limited sample size of patients receiving individual RPCHMs. Further larger scale or prospective studies are warranted to delineate the renoprotective effects of various RPCHMs.

Similar to western medicine, the use of traditional herbal medicine including* Angelica sinensis* and other RPCHMs also may have undesirable effects if they are used without careful evaluation by the treating physicians [19]. For example, ferulic acid, an extract derived from* Angelica sinensis*, has adverse immunomodulatory effects in patients with certain hormone-sensitive conditions such as breast cancer or ovarian cancer [45]. Moreover,* Astragalus membranaceus* is well known to inhibit cytochrome P450 3A4 (CYP3A4) and can reduce cyclophosphamide-induced immunosuppression [46]. Long-term use of* Rheum palmatum* had been reported to cause electrolyte depletion and liver toxicity as well [47]. Therefore, although RPCHMs may be beneficial to patients with CKD, initiation of treatment with potentially renoprotective CHMs should always be carefully evaluated by licensed Chinese medicine physicians.

Hypertension is a well-known risk factor for CKD and its progression. Poorly controlled blood pressure can lead to CKD deterioration and consequent CVD and mortality [48]. In the present study, we found that hypertension also played an important role in both all-cause mortality and CKD-related mortality. Apart from hypertension, DM is an important risk factor for CKD progression as well. According to the Diabetes Control and Complications Trial [49] and UK Prospective of Diabetes Study [50], patients with poorly controlled type 2 DM had a higher chance of developing macroalbuminuria and progressively worsened nephropathy or even death. Our findings on DM are thus consistent with previous reports.

CVD is a major complication that can cause death in patients with CKD [25]. In this study, although CVD was a risk factor for mortality in the bivariate analysis (Table 1), it was not associated with the risk of mortality after controlling for other confounding variables. This is probably because of the presence of collinearity between CKD and other comorbidities, especially hypertension, DM [48, 51], and age. Similarly, CCI, an indicator used to assess the overall burden of disease in patients [23], also lost its statistical significance in multivariate Cox proportional hazards regression models.

Poverty affects the accessibility to medical treatment for patients with CKD and related mortality [52, 53]. Although the overwhelming majority of Taiwanese residents are included in the NHI program, patients must pay a nominal out-of-pocket copayment out of their own pockets for each clinic visit. Therefore, patients from poorer economic conditions might have less medical access and poor disease control, which could then lead to a higher risk of mortality.

Smoking was found to be an attributable risk in 31% of CKD cases in a large cohort study [54]. Moreover, the risk of rapid progression to ESRD was 3.5 times higher in patients with CKD who smoked more than five packets of cigarettes per year [55]. As the information on smoking status was unavailable in the NHIRD, we employed COPD as a surrogate marker for smoking status and found a positive association between COPD and all-cause mortality. Although smoking accounts for 80 to 90% of the incidence of COPD, there are other etiologies of COPD such as air pollution, exposure to chemicals or irritating substances, dust, and allergens [56]. Therefore, the observed association between COPD and all-cause mortality may or may not completely reflect the relation between smoking and mortality.

Some prior studies suggested that the use of NSAIDs and analgesics was associated with CKD progression, the development of ESRD, or death [24, 57]. However, not all studies supported the positive association between NSAID exposure and the risk of renal function loss [58, 59]. We found the use of ≧104 pills of NSAIDs or analgesics per year was associated with all-cause mortality and CKD-related mortality in patients with incident CKD, which supports the nephrotoxic potential of both NSAIDs and analgesic therapy.

Erythropoietin is a glycoprotein hormone with the function of regulating the formation of red blood cells in the human body and is commonly used to treat anemia following dialysis for patients with ESRD. In Taiwan, the use of erythropoietin is strictly limited to patients with ESRD who have a Hct value less than 28% or patients with CKD who have a serum creatinine greater than 6 mg/dL and a Hct value less than 28%. Therefore, the use of erythropoietin was associated with the risk of all-cause mortality and CKD-related mortality. Similarly, patients who were hospitalized prior to the diagnosis of CKD were also associated with the risk of mortality.

## 5. Limitations

We found that the use of prescribed RPCHMs, especially* Angelica sinensis*, was associated with a lower risk of mortality in patients with incident CKD. However, the study findings should be interpreted with caution, and a causal relation between the use of RPCHMs and the risk of mortality cannot be inferred based on such a retrospective follow-up study because of several methodological limitations. First, in this observational study, we are unable to completely exclude the possibility of unmeasured confounding effects. For example, several potential risk factors of CKD and related mortality are not available in LHID2000, such as smoking, alcohol drinking, family history of CKD, and exercise habits. These factors can thus confound the findings if they are also associated with the use of RPCHMs. Although we had used COPD and alcoholic liver disease as surrogate markers to control for the potential confounding effects of smoking and alcohol consumption, the lack of direct measurement on relevant variables might result in residual confounding.

The validity of LHID2000 may also be of concern because the database is a claims database that is not designed for research purposes. Nevertheless, the NHI program is the only nationwide insurance program in Taiwan and covers more than 96% of the Taiwanese residents. Furthermore, the accuracy of medical information recorded in LHID2000 has been validated by both the NHI Bureau and some researchers in Taiwan [60].

The information on serum creatinine or glomerular filtration rate is unavailable in LHID2000, so we were unable to stratify the data analyses by the stages of CKD. We therefore used erythropoietin as well as vitamin D therapy as indices of the severity of CKD. We found that the use of erythropoietin but not vitamin D therapy was significantly associated with the risk of both all-cause and CKD-related mortality. We further revealed that the inverse associations between the use of RPCHMs and mortality were not present among patients with CKD who already received erythropoietin.

We initially attempted to evaluate the beneficial effects of individual RPCHMs, but numerous RPCHM formulations are prescribed in Taiwan, and it is extremely difficult to calculate the cumulative dose of each one. Moreover, the number of patients receiving most RPCHMs was relatively limited. We therefore divided the use of RPCHMs into those containing* Angelica sinensis* and those that do not. This led to a difficulty in disentangling the potential renoprotective effect across various RPCHMs. We were also unable to compare the difference in renoprotective effect between the use of* Angelica sinensis* alone and the consumption of* Angelica sinensis* with other RPCHMs because* Angelica sinensis* was almost always prescribed concomitantly with other RPCHMs in this study. Further larger scale and prospective studies are warranted to better delineate the renoprotective effects of individual RPCHM and the potential interactions between different RPCHMs.

Selection bias is still possible in this retrospective population-based follow-up study. To minimize such bias, we excluded patients who had died within the first month after the diagnosis of incident CKD because they were less likely to receive RPCHM treatment for their illness. However, unforeseen differences in the baseline characteristics between patients who received RPCHMs after CKD diagnosis and those who did not could still be present and account for the findings of this study.

Finally, information related to the use of nonprescribed CHMs is not included in LHID2000. In a previous case-control study, we found that 6.6% of the controls had received nonprescribed CHMs, and the use of nonprescribed CHMs was positively associated with the risk of CKD [10]. We speculated that such an association was probably attributable to the adulteration of nonprescribed CHMs by pharmaceuticals. In Taiwan, the NHI Bureau mandates that all reimbursed CHMs be manufactured following “Good Manufacturing Practice (GMP).” There was also no significant relationship between the use of nonprescribed and prescribed CHMs among patients with CKD in a previous study [10]. Therefore, the use of nonprescribed CHMs was less likely to contribute to the observed inverse association between RPCHMs and the risk of mortality.

## 6. Conclusions

We found that the use of certain RPCHMs after the diagnosis of CKD, especially* Angelica sinensis*, was associated with a lower risk of all-cause and CKD-related mortality among patients with incident CKD. However, such an association was not observed among patients with severe CKD who required erythropoietin therapy.

## Figures and Tables

**Table 1:** Distribution of baseline characteristics between 10,777 alive subjects and 848 deceased subjects who had incident chronic kidney disease.

Variables	Survival (*n*, %) (*n* = 10777)	Death (*n*, %)			
		All-cause (*n* = 848)	*p* value	CKD related (*n* = 279)	*p* value
Gender					
Female	4062 (37.7)	295 (34.8)	0.092	116 (41.6)	0.186
Male	6715 (62.3)	553 (65.2)		163 (58.4)	
Age (year, mean ± SD)	53.8 ± 20.8	71.8 ± 14.1	<0.001	69.9 ± 14.2	<0.001
<40	2718 (25.2)	23 (2.7)	<0.001	9 (3.2)	<0.001
40–55	2603 (24.2	89 (10.5)		39 (14.0)	
56–70	2663 (24.7)	188 (22.2)		65 (22.3)	
>70	2793 (25.9)	548 (64.6)		166 (59.5)	
Monthly wage (NTD)					
≤15840	4774 (44.3)	584 (68.9)	<0.001	186 (66.7)	<0.001
15841–19200	3764 (34.9)	220 (25.9)		79 (28.3)	
>19200	2239 (20.8)	44 (5.2)		14 (5.0)	
Hypertension	5212 (48.4)	656 (77.4)	<0.001	220 (78.9)	<0.001
Diabetes mellitus	3257 (30.2)	407 (48.0)	<0.001	148 (53.1)	<0.001
Cardiovascular disease^*∗*^	2466 (22.9)	329 (38.8)	<0.001	102 (36.6)	<0.001
CCI					
0	2633 (24.4)	94 (11.1)	<0.001	39 (14.0)	<0.001
1	2299 (21.3)	131 (15.5)		37 (13.2)	
≧2	5845 (54.2)	623 (73.4)		203 (72.8)	
Ever being hospitalized before CKD	1009 (9.4)	216 (25.5)	<0.001	109 (39.1)	<0.001
COPD	1540 (14.3)	278 (32.8)	<0.001	76 (27.2)	<0.001
Alcoholic liver disease	1409 (13.1)	132 (15.6)	0.039	47 (16.9)	0.066
Use of analgesics or NSAIDs > 104 pills/year	373 (3.5)	53 (6.3)	<0.001	15 (5.4)	0.086
Receipt of erythropoietin	539 (5.0)	97 (11.4)	<0.001	52 (18.6)	<0.001
Receipt of vitamin D	109 (1.0)	12 (1.4)	0.264	6 (2.1)	0.071
Current RPCHM use					
None	8936 (82.9)	770 (90.8)	<0.001	253 (90.7)	0.004
Other RPCHMs	513 (4.8)	25 (3.0)		9 (3.2)	
*Angelica sinensis*	1328 (12.3)	53 (6.2)		17 (6.1)	
Ever use of RPCHMs	2046 (19.0)	127 (15.0)	0.004	41 (14.7)	0.070

CCI: Charlson's Comorbidity Index; CKD: chronic kidney disease; COPD: chronic obstructive pulmonary disease; NSAIDs: nonsteroidal anti-inflammatory drugs; NTD: New Taiwan dollars; RPCHMs: renoprotective Chinese herbal medicines. ^*∗*^Including congenital heart disease.

**Table 2:** Multivariate Cox proportional hazards regression analysis of all-cause and CKD-related mortality of 11,625 CKD patients.

Variables	All-cause mortality			CKD related mortality		
	HR	95% CI	*p* value	HR	95% CI	*p* value
Gender (male)	1.2	1.1–1.4	0.007	1.0	0.8–1.3	0.937
Age (year)						
<40	Reference	—	—	Reference	—	—
40–55	4.3	2.7–6.9	<0.001	4.2	2.0–8.9	<0.001
56–70	6.4	4.1–10.0	<0.001	4.6	2.2–9.6	<0.001
>70	14.7	9.4–23.0	<0.001	10.1	4.9–20.8	<0.001
Monthly wage (NTD)						
≤15840	Reference	—	—	Reference	—	—
15841–19200	0.6	0.5–0.7	<0.001	0.6	0.5–0.8	<0.001
>19200	0.4	0.3–0.5	<0.001	0.3	0.2–0.6	<0.001
Hypertension	1.4	1.2–1.7	<0.001	1.6	1.2–2.2	0.004
Diabetes mellitus	1.2	1.1–1.4	0.005	1.3	1.0–1.7	0.027
Ever being hospitalized before CKD	2.5	2.1–2.9	<0.001	4.5	3.5–5.8	<0.001
COPD	1.2	1.0–1.4	0.014	—	—	—
Use of NSAIDs or analgesics > 104 pills/year	1.8	1.4–2.4	<0.001	1.7	1.0–2.9	0.043
Receipt of erythropoietin	1.4	1.1–1.7	0.007	1.8	1.3–2.5	<0.001
Current RPCHM use						
None	Reference	—	—	Reference	—	—
Other RPCHMs	0.6	0.4–0.9	0.013	0.7	0.4–1.4	0.346
*Angelicasinensis*	0.6	0.4–0.8	<0.001	0.6	0.4–0.9	0.025
Ever use of PRCHMs	1.0	0.8–1.2	0.790	0.9	0.7–1.3	0.722

CCI: Charlson's Comorbidity Index; CI: confidence interval; CKD: chronic kidney disease; COPD: chronic obstructive pulmonary disease; HR: hazard ratio; NTD: New Taiwan dollars; RPCHMs: renoprotective Chinese herbal medicines.

**Table 3:** Stratified multivariable Cox proportional hazards regression analysis of all-cause and CKD related mortality of 11,625 CKD patients.

Subgroups	All-cause mortality				CKD-related mortality			
	Other RPCHMs		*Angelica sinensis*		Other RPCHMs		*Angelica sinensis*	
	HR (95% CI)	*p*	HR (95% CI)	*p*	HR (95% CI)	*p*	HR (95% CI)	*p*
*Age (years)* ^∗^								
<56	0.4 (0.1–1.2)	0.085	0.5 (0.3–0.9)	0.023	—	—	0.4 (0.2–1.1)	0.089
≧56	0.6 (0.4–0.9)	0.008	0.5 (0.3–0.6)	<0.001	0.7 (0.4–1.4)	0.336	0.4 (0.2–0.8)	0.006
*Gender*								
Male	0.5 (0.3–0.8)	0.003	0.4 (0.3–0.6)	<0.001	0.3 (0.1–0.9)	0.030	0.3 (0.2–0.6)	0.001
Female	0.4 (0.2–0.8)	0.009	0.3 (0.2–0.4)	<0.001	0.7 (0.4–1.7)	0.523	0.4 (0.2–0.7)	0.004
*Hypertension*								
No	0.3 (0.1–0.8)	0.020	0.3 (0.2–0.5)	<0.001	0.2 (0.1–1.6)	0.131	0.2 (0.1–0.7)	0.008
Yes	0.5 (0.3–0.9)	0.007	0.5 (0.4–0.7)	<0.001	0.6 (0.3–1.3)	0.230	0.5 (0.3–0.9)	0.024
*Diabetes mellitus*								
No	0.4 (0.2–0.7)	0.002	0.4 (0.3–0.5)	<0.001	0.4 (0.2–1.2)	0.099	0.4 (0.2–0.8)	0.006
Yes	0.6 (0.3–1.0)	0.053	0.4 (0.3–0.6)	<0.001	0.6 (0.3–1.5)	0.311	0.3 (0.1–0.7)	0.006
*Receipt of erythropoietin*								
No	0.4 (0.3–0.6)	<0.001	0.3 (0.2–0.4)	<0.001	0.3 (0.1–0.8)	0.012	0.2 (0.1–0.4)	<0.001
Yes	1.0 (0.4–2.4)	0.952	1.2 (0.7–2.3)	0.485	1.6 (0.6–4.4)	0.399	1.7 (0.8–3.6)	0.175
*Use of NSAID or analgesics > 104 pills/year* ^∗^								
No	0.4 (0.3–0.7)	<0.001	0.4 (0.3–0.5)	<0.001	0.5 (0.3–1.0)	0.045	0.3 (0.2–0.6)	<0.001
Yes	1.1 (0.3–4.4)	0.930	0.2 (0.1–1.2)	0.081	—	—	0.7 (0.9–5.1)	0.689
*Ever being hospitalized before CKD*								
No	0.5 (0.3–0.7)	<0.001	0.4 (0.3–0.5)	<0.001	0.6 (0.3–1.3)	0.185	0.4 (0.2–0.7)	0.001
Yes	0.6 (0.3–1.6)	0.330	0.3 (0.1–0.6)	0.002	0.5 (0.1–2.1)	0.359	0.4 (0.2–1.0)	0.052

*Total study subjects*	*0.6 (0.4*–*0.9)*	*0.013*	*0.6 (0.4*–*0.8)*	*<0.001*	*0.7 (0.4*–*1.4)*	*0.346*	*0.6 (0.4*–*0.9)*	*0.025*

CI: confidence interval; CKD: chronic kidney disease; HR: hazard ratio; RPCHMs: renoprotective Chinese herbal medicines. ^*∗*^The hazard ratio for certain subgroups could not be obtained because of too small sample size.
